# Estrogen modulation of cortical spreading depression

**DOI:** 10.1186/s10194-023-01598-x

**Published:** 2023-05-26

**Authors:** Chiho Kudo, Andrea M. Harriott, Michael A. Moskowitz, Christian Waeber, Cenk Ayata

**Affiliations:** 1 Neurovascular Research Unit, Department of Radiology, Massachusetts General Hospital, Harvard Medical School, Charlestown, MA 02129 USA; 2 Department of Dental Anesthesiology, Osaka University Graduate School of Dentistry, Suita, Osaka 5650871 Japan; 3 Stroke Service, Department of Neurology, Massachusetts General Hospital, Harvard Medical School, Charlestown, MA 02129 USA; 4 School of Pharmacy, University College Cork, Cork, Ireland; 5 Department of Pharmacology and Therapeutics, University College Cork, Cork, Ireland

**Keywords:** Gonadal hormones, Migraine, Aura, Estrogen replacement, Cortical spreading depression

## Abstract

**Background and aims:**

Cortical spreading depression (CSD), a transient neuronal and glial depolarization that propagates slowly across the cerebral cortex, is the putative electrophysiological event underlying migraine aura and a headache trigger. Migraine is three times more prevalent in women than men, linked to circulating female hormones. High estrogen levels or estrogen withdrawal may be a migraine trigger for many women. We, therefore, aimed to examine whether sex, gonadectomy, and female hormone supplementation and withdrawal affect the susceptibility to CSD.

**Methods:**

To determine CSD susceptibility, we recorded the frequency of CSDs triggered during 2-h topical KCl application in intact or gonadectomized female and male rats, without or with estradiol or progesterone supplementation via daily intraperitoneal injections. Estrogen or progesterone treatment followed by withdrawal was studied in a separate cohort. To take the first step towards identifying potential mechanisms, we studied glutamate and GABA_A_ receptor binding using autoradiography.

**Results:**

The CSD frequency in intact female rats was higher than intact male and ovariectomized rats. We did not detect a change in CSD frequency during different stages of the estrous cycle in intact females. Daily estrogen injections for three weeks did not change CSD frequency. However, one-week estrogen withdrawal after two weeks of treatment significantly increased CSD frequency compared with the vehicle group in gonadectomized females. The same protocol of estrogen treatment and withdrawal was ineffective in gonadectomized males. In contrast to estrogen, daily progesterone injections for three weeks elevated CSD susceptibility, and one-week withdrawal after two weeks of treatment partially normalized this effect. Autoradiography did not reveal significant changes in glutamate or GABA_A_ receptor binding density after estrogen treatment and withdrawal.

**Conclusions:**

These data suggest that females are more susceptible to CSD, and sexual dimorphism is abrogated by gonadectomy. Moreover, estrogen withdrawal after prolonged daily treatment enhances CSD susceptibility. These findings may have implications for estrogen-withdrawal migraine, although the latter tends to be without aura.

**Supplementary Information:**

The online version contains supplementary material available at 10.1186/s10194-023-01598-x.

## Introduction

Migraine is the most common neurological condition observed in approximately 12–15% of the population, and its prevalence is almost 3 times higher among women than men [1, 2]. This difference starts after puberty and disappears after menopause, peaking between ages 20 and 40, suggesting modulation by female hormones [3]. Migraine attacks can also be influenced by hormonal fluctuations, especially during the female menstrual cycle [4–6]. Moreover, female migraine patients who take oral contraceptives may experience more severe migraine attacks during hormone-free intervals, defined as “estrogen-withdrawal migraine”. Altogether, the data suggest that fluctuations in estrogens modulate susceptibility to migraine attacks in women, but the mechanism of this modulation is unknown.

Cortical spreading depression (CSD) is a wave of neuronal and glial depolarization that slowly propagates throughout the cortex irrespective of functional divisions or vascular territories [7]. A link between CSD and migraine aura has been hypothesized for more than 40 years [8–10] and is strongly supported by clinical and experimental data [11–14]. Auras often precede the headache, and experimental data show that CSD can activate the trigeminovascular system resulting in headache [15–17]. Since prophylactic drugs that are clinically effective in migraine without aura also suppress CSD [18], the latter may also play a role in the pathophysiology of migraine without aura [14]. However, it is not yet clear how CSD is triggered in migraine patients, although ischemic or epileptic mechanisms have been implicated [19, 20]. Many factors (e.g., genetic, hormonal, environmental) modulate the susceptibility to CSD [21–29]. Positive interactions among these factors have also been described in familial hemiplegic migraine type 1 knockin mice [21, 30].

Our study aimed to examine the effect of sex, gonadectomy, and prolonged daily treatment with female hormones and their withdrawal, mimicking daily oral contraceptive use, on CSD susceptibility using a well-established experimental model [18, 31].

## Materials and methods

### Animals

Experiments were approved by the MGH Institutional Animal Care and Use Committee and carried out per the Guide for Care and Use of Laboratory Animals (NIH Publication No. 85–23, 1996). Rats (Sprague-Dawley, 250–400 g, male and female, intact or gonadectomized; Charles River Laboratories, Wilmington, MA, USA) were housed in a climate-controlled environment and a dark/light cycle of 12 h of daylight and 12 h of darkness with ad libitum access to food and water.

### CSD susceptibility

#### Surgical preparation

Rats were anesthetized using isoflurane (3% for induction, 2% for maintenance during surgical procedures). Bupivacaine (0.25%) was administered locally at surgical sites for analgesia before the incisions. Rats were intubated via a tracheostomy for mechanical ventilation (SAR-830; CWE, Ardmore, PA, USA). Continuous measurement of mean arterial blood pressure (PowerLab; ADInstruments, Colorado Springs, MO, USA) and arterial blood sampling was performed via a femoral artery catheter. Rats were then paralyzed by intraperitoneal pancuronium (0.32–0.40 mg/kg/h) to facilitate mechanical ventilation. Arterial blood gases and pH were measured every 15–30 min and ventilation parameters were adjusted to maintain the arterial pCO_2_ at 35–45 mmHg (Corning 178 blood gas/pH analyzer; Corning, NY, USA). Rectal temperature was kept at 36.9 to 37.1 °C using a thermostatically controlled heating pad (Frederick Heat Company, Bowdoinham, ME, USA). The level of anesthesia was maintained throughout the procedure to abolish the blood pressure and the heart rate response to tail pinch. Adequate measures were taken to minimize pain or discomfort. Mean arterial blood pressure, pCO_2_, pO_2_ and pH were within physiological range for anesthetized and artificially ventilated animals (Table 1) [32].

**Table 1:** Systemic physiology

		Body weight (g)	pH	pCO2 (mmHg)	pO2 (mmHg)	Blood pressure (mmHg)
Exp 1	Male	400 ± 18	7.42 ± 0.00	38 ± 1	144 ± 3	98 ± 3
	Female	310 ± 5	7.43 ± 0.01	35 ± 1	145 ± 3	121 ± 3
	Ovx	353 ± 11	7.44 ± 0.00	37 ± 1	148 ± 3	113 ± 4
Exp 2	Estrus	324 ± 9	7.43 ± 0.01	34 ± 1	142 ± 5	121 ± 5
	Proestrus	307 ± 8	7.43 ± 0.01	35 ± 2	150 ± 3	126 ± 4
	Metestrus/ diestrus	323 ± 11	7.41 ± 0.01	39 ± 1	150 ± 5	125 ± 6
Exp 3	Ovx + V	342 ± 5	7.45 ± 0.01	38 ± 1	175 ± 2	107 ± 4
	Ovx + E	283 ± 7	7.42 ± 0.01	39 ± 1	172 ± 4	109 ± 5
	Ovx + E/W	285 ± 5	7.45 ± 0.00	37 ± 0	172 ± 4	121 ± 9
Exp 4	Cst + V	401 ± 8	7.44 ± 0.00	37 ± 0	168 ± 6	103 ± 3
	Cst + E	308 ± 9	7.43 ± 0.01	36 ± 1	166 ± 4	96 ± 3
	Cst + E/W	360 ± 9	7.43 ± 0.01	38 ± 1	173 ± 3	105 ± 4
Exp 5	Ovx + P	349 ± 5	7.45 ± 0.01	36 ± 1	166 ± 13	114 ± 3
	Ovx + P/W	329 ± 15	7.44 ± 0.01	39 ± 2	179 ± 2	119 ± 4

**Fig. 1:**
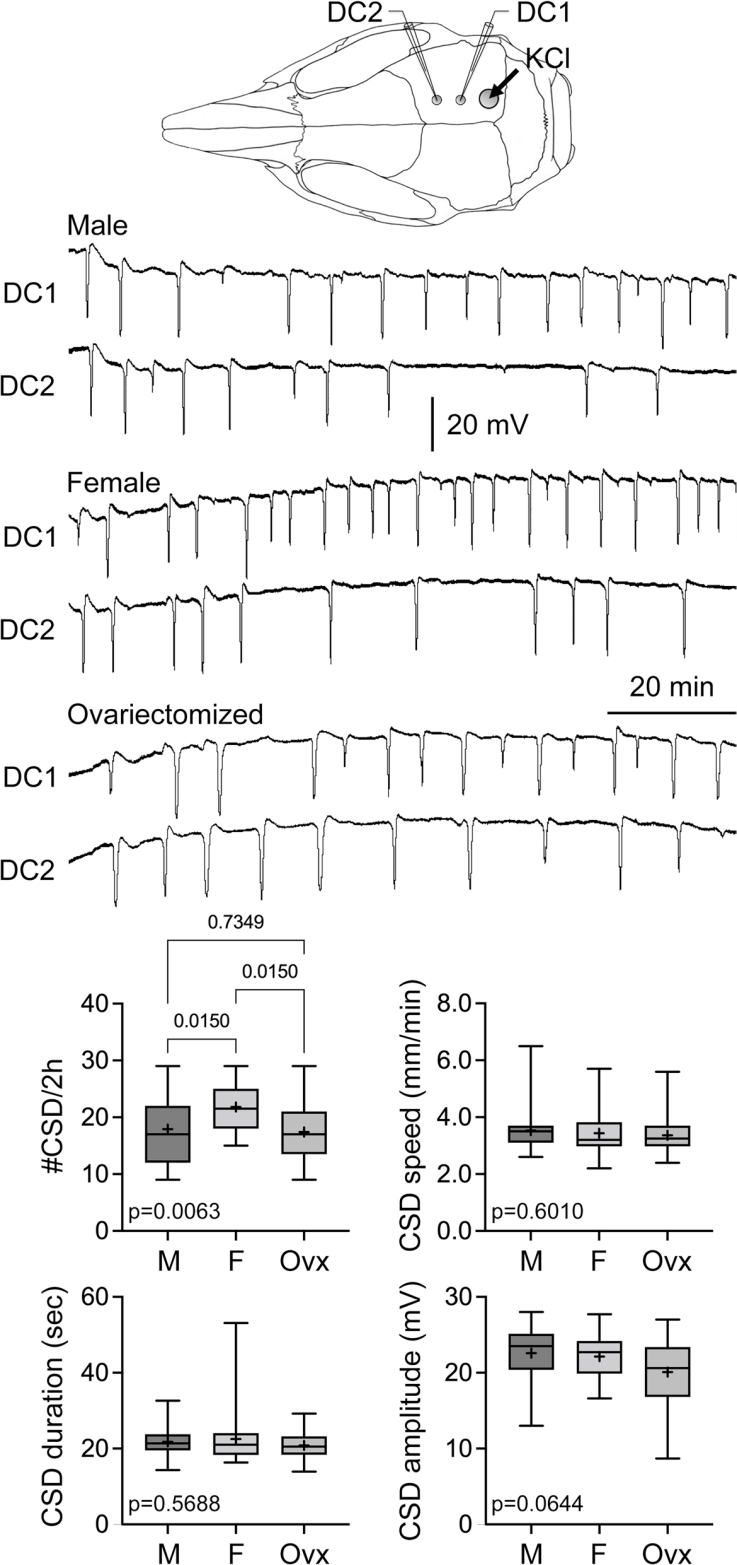
Effect of female sex and ovariectomy on CSD susceptibility. Representative DC potentials from DC1 and DC2 recording sites show recurrent CSDs triggered by topical 1 M KCl application onto the occipital cortex. CSD frequency, speed, duration, and amplitude are shown for male (*n* = 29), female (*n* = 26) and ovariectomized females (*n* = 21)

**Fig. 2:**
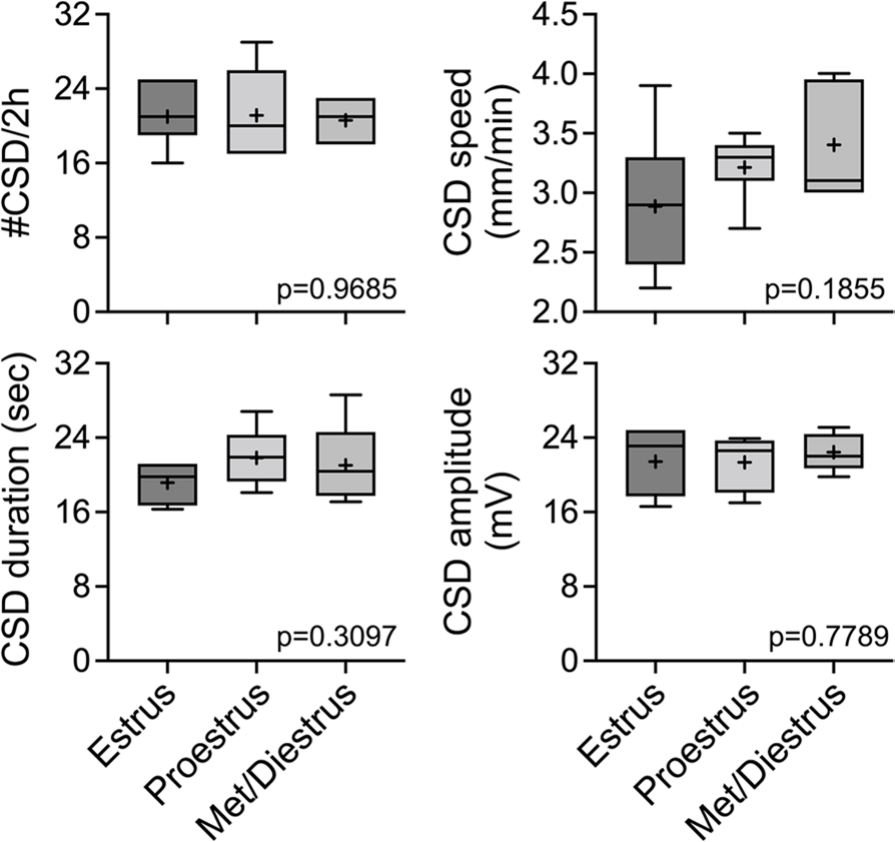
The estrous cycle stages did not differ in CSD susceptibility. There was no difference among the estrus (*n* = 7), proestrus (*n* = 7), and metestrus/diestrus (*n* = 5) stages in CSD frequency, speed, duration, or amplitude triggered by topical 1 M KCl as shown in Fig. 1. Data shown here are from the female group in Fig. 1

**Fig. 3:**
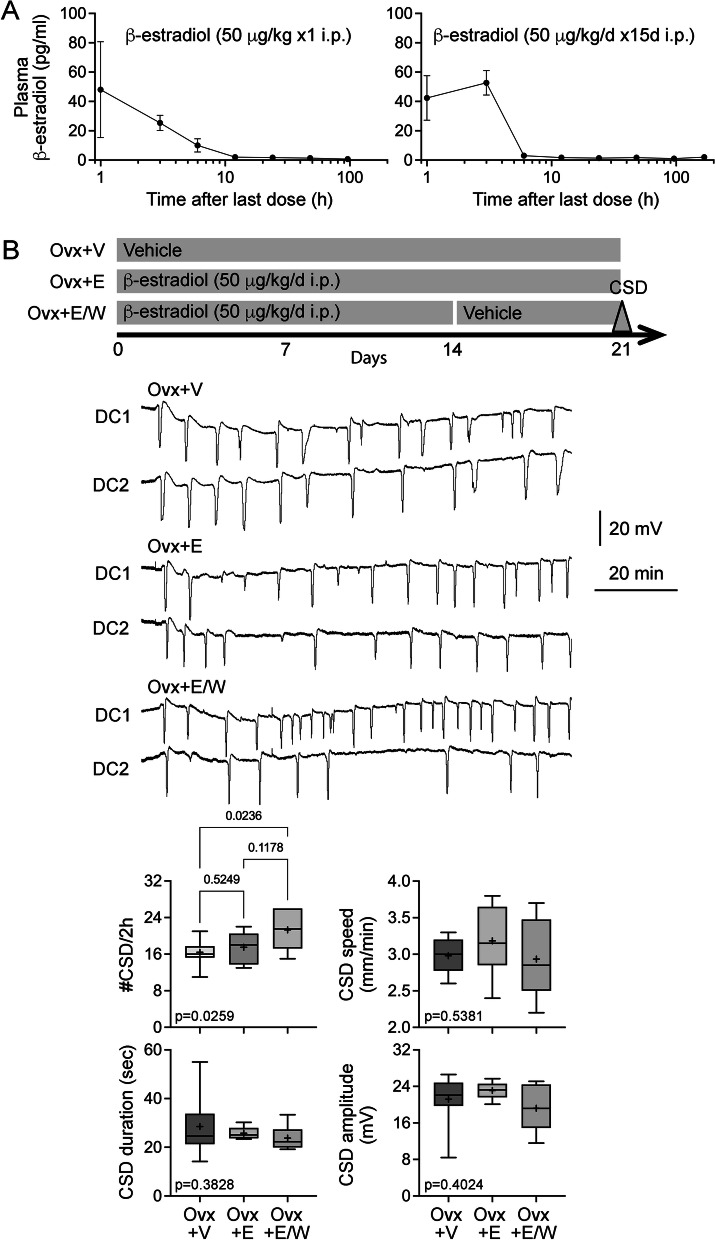
Effects of estradiol replacement and withdrawal in ovariectomized rats on CSD susceptibility. **A** Plasma concentrations of β-estradiol after a single intraperitoneal injection (left panel) and once-daily intraperitoneal injection (right panel) of 50 μg/kg/day β-estradiol-3-benzoate. Samples were collected at indicated times after the last dose. **B** Experimental timeline shows the treatment protocol with once-daily intraperitoneal injections in ovariectomized rats. Representative DC potentials from DC1 and DC2 recording sites show recurrent CSDs triggered by topical 1 M KCl application onto the occipital cortex, as shown in Fig. 1. CSD frequency, speed, duration, and amplitude are shown for ovariectomized rats after 3 weeks of vehicle treatment (Ovx + V, *n* = 12), ovariectomized rats after 3 weeks of 50 μg/kg/day β-estradiol-3-benzoate treatment (Ovx + E, *n* = 6) and ovariectomized rats treated with 50 μg/kg/day β-estradiol-3-benzoate for 2 weeks followed by withdrawal (Ovx + E/W, *n* = 6)

**Fig. 4:**
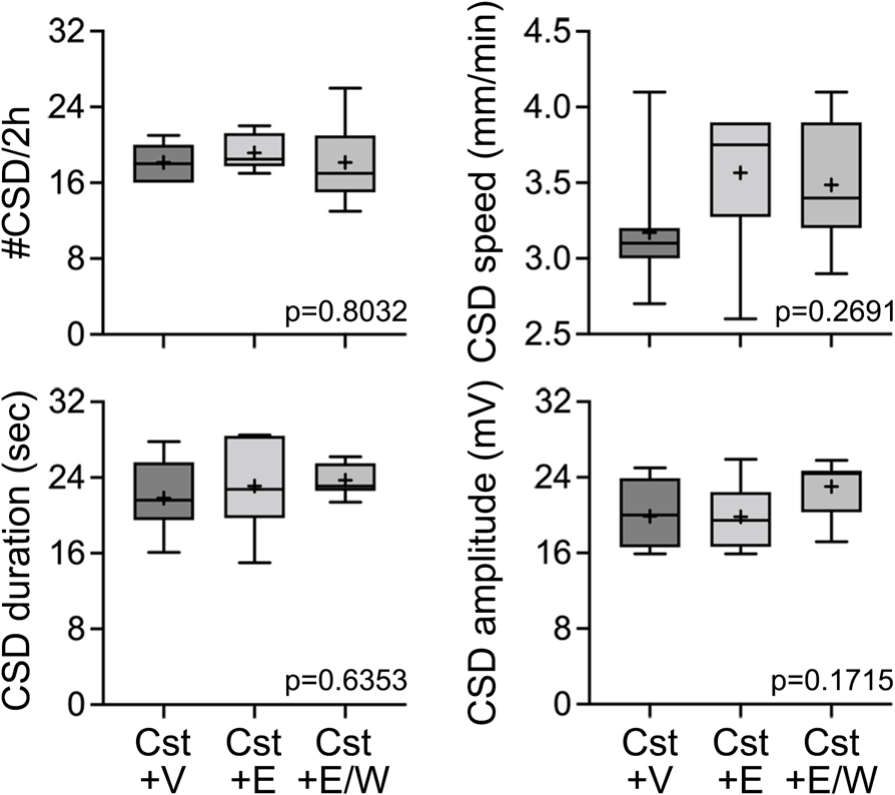
Effects of estradiol replacement and withdrawal in castrated rats on CSD susceptibility. CSD frequency, speed, duration, and amplitude are shown for castrated rats after 3 weeks of vehicle treatment (Cst + V, *n* = 7), castrated rats after 3 weeks of 50 μg/kg/day β-estradiol-3-benzoate treatment (Cst + E, *n* = 6) and castrated rats treated with 50 μg/kg/day β-estradiol-3-benzoate for 2 weeks followed by withdrawal (Cst + E/W, *n* = 7)

Experiments (Exp) 1–4 are shown on Figs. 1, 2, 3 and 4, respectively. Exp 5 is shown on Fig. S1

#### Electrophysiological recordings

Rats were placed in a stereotaxic frame (David Kopf Instruments, Tujunga, CA, USA) and three burr holes were drilled under saline cooling over the right hemisphere at the following coordinates (mm from bregma): (1) posterior 5, lateral 2 (occipital cortex, 2 mm diameter for KCl application); (2) posterior 3.5, lateral 2 (parietal cortex, 1 mm diameter, recording site 1); (3) posterior 1, lateral 2 (frontal cortex, 1 mm diameter, recording site 2). At the KCl application hole, dura overlying the occipital cortex was gently removed and care was taken to avoid cortical damage or bleeding, which was an exclusion criterion. The steady (DC) potential and the electrocorticogram were recorded with glass micropipettes filled with 150 mM NaCl, inserted 300 μm below the dural surface (FHC, Inc. Bowdoinham, ME, USA). The Ag/AgCl reference electrode was placed subcutaneously in the neck. After the surgical preparation, the cortex was allowed to recover for 15 min under saline irrigation. The data were continuously recorded using a data acquisition system for offline analysis.

#### CSD induction

A cotton ball (2 mm diameter) soaked with 1 M KCl was placed on the pial surface and kept moist by placing 5 μl of KCl solution every 15 min. The number of KCl-induced CSDs was counted for 2 h. Propagation speed was calculated from the distance and CSD latency between the recording electrodes 1 and 2. The DC shift amplitude and duration at half-maximal amplitude were also measured. We used the frequency of KCl-induced CSDs (per 2 h) as the primary readout for CSD susceptibility because it generally correlates with CSD threshold measured by other means (e.g., escalating cathodal stimulation [12]), and because CSD frequency yields data with a normal distribution. The propagation speed, duration, and amplitude do not directly relate to CSD susceptibility and are used as important quality control measures (e.g., abnormal systemic physiology, local tissue damage, poor electrode positioning).

### Vaginal cytology

To determine the specific estrous stage, vaginal smears were collected by an eyedropper and 0.9% saline. The eyedropper's tip was filled with a small amount of saline and inserted into the vagina. The fluid was expelled into the vagina, collected, placed onto a slide, and examined under a light microscope (× 10–20 magnification), while still wet [33]. Stages were tracked for at least two complete estrous cycles before the experiments to capture a relatively even number of animals in each stage. The final estrous stage determination was performed immediately before anesthesia induction and approximately 80–120 min before CSD recordings, as previously described [23, 25].

### Hormone replacement

At least 6 days after gonadectomy, we started treatment with daily intraperitoneal injections of vehicle (0.2 ml sesame oil, *n* = 12 females, 7 males), β-estradiol-3-benzoate (50 μg/kg/day dissolved daily in sesame oil, *n* = 6 females, 6 males; Sigma, Saint Louis, MO, USA), or progesterone [34] (5 mg/kg/day dissolved daily in sesame oil, *n* = 5 females; Sigma, Saint Louis, MO, USA) for 21 days in gonadectomized (ovariectomy, Ovx; castration, Cst) rats. In a third group, after 14 days of treatment, either hormone was replaced with the vehicle for 7 days (estrogen withdrawal, *n* = 6 Ovx, 7 Cst; progesterone withdrawal, *n* = 6 Ovx).

### Enzyme immunoassay for plasma estradiol measurement

Rats were treated with a single or 15-day once-daily intraperitoneal β-estradiol-3-benzoate (50 μg/kg) injection. After the last injection, blood was collected from the femoral artery or the jugular vein at various time points. Blood samples were collected into Microtainer (BD, Franklin Lakes, NJ, USA), centrifuged at 2700 rcf at 4 °C, and plasma was collected and stored at -80 °C. Plasma concentrations were measured by using Estradiol (E2) Enzyme Immunoassay Test Kit (BioCheck Inc, Foster City, CA, USA). Duplicate or triplicate 25 μl plasma samples were measured using a spectrophotometer according to the manufacturer’s specifications.

### Receptor autoradiography

At the end of the electrophysiological recordings, female rats (*n* = 6 per treatment group) were decapitated while under anesthesia, their brains were rapidly removed and frozen in isopentane chilled to -30 °C in dry ice. Some brains were also harvested from rats that did not undergo electrophysiological recording at the end of the treatment period. Brains were stored at  -80 °C until sectioned. Ten micron-thick sagittal sections were cut using a cryostat microtome (Microm HM505E, Thermo Fisher Scientific, Waltham, MA, USA) and thaw-mounted onto Superfrost Plus glass slides (Fisher Scientific, Pittsburgh, PA, USA) and kept at  -25 °C until used.

Aspartate, AMPA, kainate, NMDA, and GABA_A_ binding sites were visualized by quantitative in vitro receptor autoradiography using D-[^3^H]aspartate (TRK-606, 28 Ci/mmol, GE Healthcare), [^3^H]kainate (NET-875, 47 Ci/mmol, PerkinElmer), [^3^H]AMPA (NET-833, 52 Ci/mmol, PerkinElmer), [^3^H]MK-801 (ART-661, 17–25 Ci/mmol, American Radiolabeled Chemicals) and [^3^H]muscimol (NET-574, 14.7 Ci/mmol, PerkinElmer). Specific parameters for each radioligand are listed in Supplemental Table 1, but the general steps were similar. Briefly, sections were brought to room temperature 15 min before preincubation in a radioligand-free buffer (to reduce the levels of endogenous ligands and modulators). Sections were then incubated with nanomolar concentrations of tritiated ligands in the absence (total binding) or in the presence of an excess of unlabeled drug specific for the target receptor (to assess nonspecific binding). After washing steps in ice-cold buffer (to remove unbound radioligand) and a rinse in ice-cold deionized water (to remove salts) or fixation step in 2.5% glutaraldehyde, sections were dried under a stream of cold air and exposed for 1 to 2 weeks to a tritium sensitive film (Amersham ^3^H-Hyperfilm, Arlington Heights, IL, USA) in a light-tight X-ray cassette. After film processing, radioligand binding in selected brain regions was assessed by measuring the optical density of the autoradiograms using a computerized image analysis system (Imaging Research, St. Catharines, Ontario, Canada). The density of specific labeling was obtained by subtracting nonspecific binding from total binding values.

### Statistical analysis

Data were expressed as whisker-box plots or mean ± standard error of the mean (SEM). Analyses were made using Prism 9 (Graphpad Software, Inc., CA, USA). Individual statistical tests are indicated in text or figure legends. *P* < 0.05 was considered statistically significant. All data were analyzed in a blinded fashion. The CSD frequency was the primary endpoint in all experiments, on which sample size calculations were based. In the absence of prior experience with gonadal hormone effects on CSD frequency in rats in our lab, the initial sample size calculation empirically targeted 90% power to detect 40% effect size, assuming a coefficient of variation of 25%. After completion of the experiments to reach this sample size, we performed a second power calculation, using the observed effect size and the coefficient of variation in this pilot cohort. If the updated sample size needed to achieve statistical significance was more than 20, we deemed it futile and stopped the experiments. Otherwise, we resumed the experiments to reach the updated sample size. Because experiments were spread over 2 years, we frequently included additional untreated rats as time controls during the later experiments and combined them with the initial cohorts studied; therefore, the sample sizes in the earlier experiments were larger than in the subsequent experiments. Experimental losses due to technical failures also contributed to the variation in sample sizes in different groups and cohorts. All sample sizes and exclusions are indicated in the figure legends. The normal distribution of data was confirmed using the Shapiro-Wilk test for each dataset. Normally distributed data were analyzed using one-way ANOVA followed by Holm-Sidak multiple comparisons and expressed as whisker-box plots. Non-normal distributed data were analyzed using the Kruskal-Wallis test.

## Results

Topical application of 1 M KCl-evoked repetitive CSDs, detected sequentially in serially placed electrodes (Fig. 1), the frequency of which was used as a previously validated measure of CSD susceptibility [35]. The CSD frequency was higher in female rats compared with males, and ovariectomy decreased CSD frequency to the level of males. None of the other CSD attributes differed among the groups. Within the intact female cohort, we did not find a difference in CSD frequency among metestrus/diestrus with low estrogen, proestrus with high estrogen, and estrus with a drop in estrogen (Fig. 2) [36]. These data suggested that gonadal hormones enhance CSD susceptibility in normal cycling females regardless of cycle stage.

We next examined the effect of daily estrogen injections in Ovx rats. First, we measured the plasma levels of β-estradiol after either a single dose or a once-daily dose for 15-days of 50 μg/kg/day intraperitoneal β-estradiol-3-benzoate. Samples were collected at various time points after the last injection. After either a single dose or 15-day treatment, plasma β-estradiol dropped to undetectable levels within 12 h (Fig. 3A). These data showed that intraperitoneal dosing results in transient daily surges rather than steady-state levels. Once-daily β-estradiol-3-benzoate (50 μg/kg/day intraperitoneal) for 21 days did not change CSD frequency; however, withdrawal of estrogen after 14 days significantly elevated CSD frequency compared with the vehicle group (Fig. 3B). Interestingly, the same treatment paradigms did not influence CSD frequency in Cst rats suggesting an interaction with sex (Fig. 4). There was no effect of β-estradiol-3-benzoate treatment or withdrawal on the other CSD attributes (Fig. 3B and 4). Altogether, these data revealed a CSD-enhancing effect of estrogen withdrawal in a prolonged daily dosing paradigm in Ovx rats. Unlike estrogen, once-daily progesterone (5 mg/kg/day intraperitoneal) for 21 days significantly elevated CSD frequency compared with vehicle in Ovx rats. Progesterone withdrawal for 1 week after 14-day treatment reversed this effect (Fig. S1). These data suggested that the effect on CSD susceptibility is hormone-specific.

**Fig. 5:**
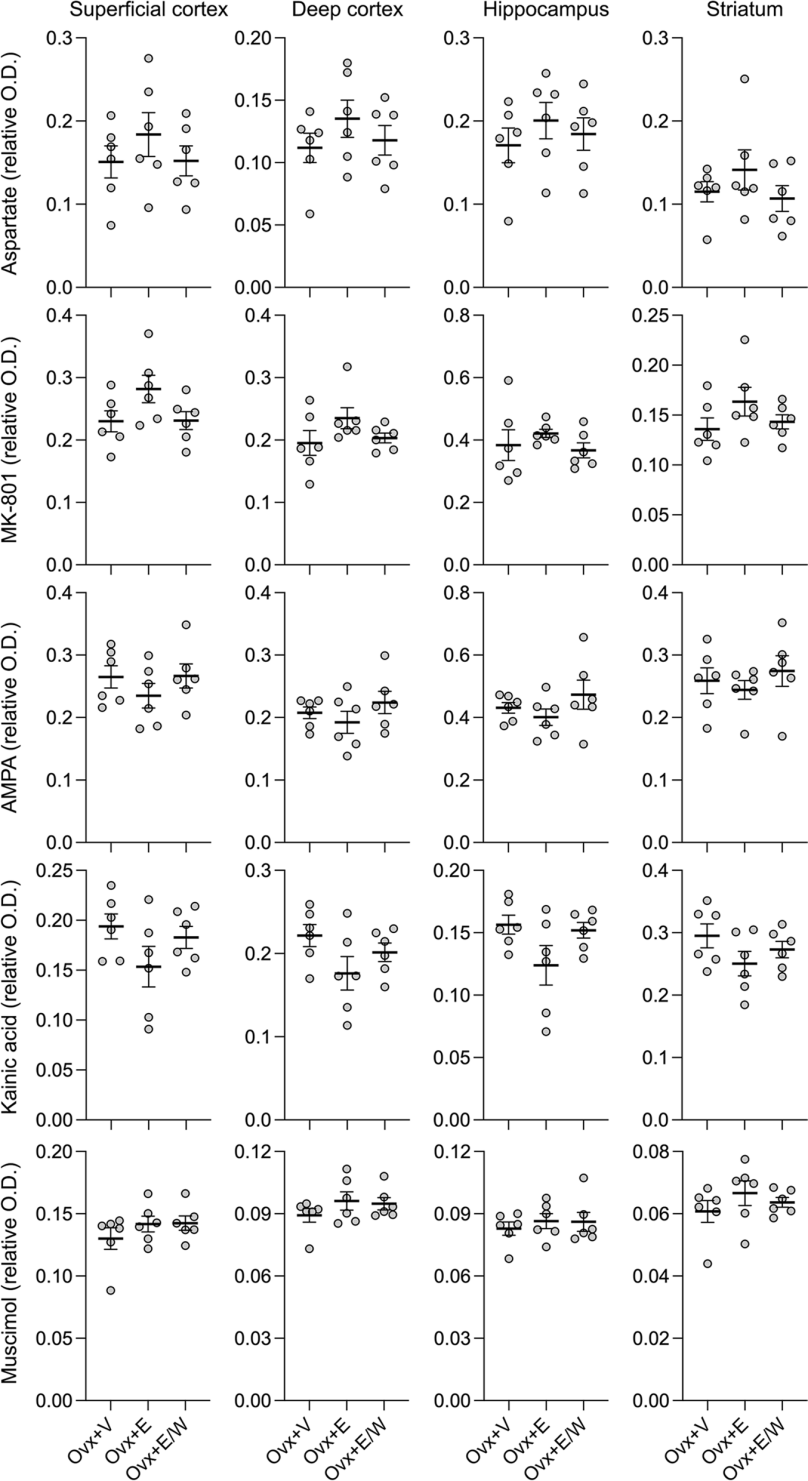
Receptor autoradiography after estrogen treatment or withdrawal. Aspartate, AMPA, kainate, NMDA, and GABA_A_ binding sites were visualized by quantitative in vitro receptor autoradiography using D-[^3^H]aspartate, [^3^H]kainate, [^3^H]AMPA, [^3^H]MK-801 and [.^3^H]muscimol in frozen sections from ovariectomized female rats treated with vehicle (Ovx + V), with β-estradiol-3-benzoate (Ovx + E), or with β-estradiol-3-benzoate followed by withdrawal (Ovx + E/W). Specific radioligand binding in selected brain regions (superficial and deep cortical layers, in the striatum and in hippocampus (CA1, CA2, CA3) was assessed by measuring the optical density of the autoradiograms using a computerized image analysis system (Imaging Research, St. Catharines, Ontario, Canada). No significant treatment effect was noted for any of the radioligands. Individual values for each rat (expressed as Relative Optical Densities) are shown, together with group means and SEMs (*n* = 6 per treatment group)

Lastly, we studied glutamate and GABA_A_ receptor density using autoradiography. The overall distribution of aspartate, AMPA, kainate, NMDA, and GABA_A_ binding sites matched the distributions previously reported. We did not find a significant difference in labeling intensity among the Ovx rats treated with vehicle, Ovx treated with estrogen, and Ovx treated with estrogen followed by withdrawal in any of the brain regions examined (superficial and deep cortex, striatum, hippocampus; Fig. 5).

## Discussion

We here show that CSD susceptibility displays sex differences and that female rats have a higher susceptibility to CSD than males. These data are consistent with a previous report in rats using comparable methods [23]. We have previously reported a similar phenotype in familial hemiplegic migraine type 1 knockin but not in wild-type mice [21]. However, others have observed a higher CSD susceptibility in female wild-type mice, which was independent of the estrous cycle [37], using methods that may have achieved a higher sensitivity [38]. Lastly, the extracellular threshold [K^+^]_e_ for eliciting CSD was lower in female rats compared with males [29]. Altogether, we believe these data establish sexual dimorphism in CSD susceptibility.

The lack of variation in CSD susceptibility among the estrous cycle stages in the rat was not surprising given that the total duration of the rat estrous cycle is 4–5 days, compared with the ~ 28-day human menstrual cycle. In the rat, metestrus/diestrus lasts 54–80 h, proestrus 12–14 h, and estrus 25–27 h. Performing the experiments at a precise time within the intended estrous cycle stage is difficult when estrogen levels can peak or trough within a just few hours [39, 40]. Moreover, the effect, as well as the mechanisms of gonadal hormone influence on CSD susceptibility, may be different on a shorter time scale. Prior work in rats using the same method also failed to detect a difference in CSD susceptibility among estrous cycle stages [23, 25]. Others have reported a lower KCl-concentration threshold to trigger CSD in mice during diestrus (i.e., enhanced CSD susceptibility). Interestingly, however, estradiol and progesterone levels measured during the diestrus were 10-fold higher and 3-fold lower, respectively, compared with the estrus stage in that study [26]. Moreover, the endpoint of KCl-concentration threshold to trigger a CSD differs from the frequency of CSDs triggered by continuous topical KCl application used herein [35]. Therefore, a direct comparison between this study and ours is difficult.

The fact that Ovx abrogated the sex difference in CSD frequency indicates that female gonadal hormones modulate CSD susceptibility. However, our daily estrogen replacement paradigm in Ovx rats did not alter KCl-induced CSD frequency, suggesting that simple daily estrogen pulses are not sufficient to reproduce the effect of functioning female gonads on CSD (Fig. 3). In contrast, withdrawal after 2 weeks of daily treatment resulted in elevated CSD frequencies. These data appeared at odds with prior work from one group in a similar rat model where the implantation of 17β-estradiol-releasing capsules for two weeks, aiming for steady serum levels corresponding to proestrus peak, increased KCl-induced CSD frequency [23, 27]. Conversely, withdrawing the 17β-estradiol-releasing capsules resulted in a frequency reduction in that study [23]. Importantly, however, the therapeutic paradigm significantly differed between our study and Chauvel et al. (i.e., daily estrogen injections vs. continuous estrogen-releasing capsules and 7-day vs. 20-h withdrawal, respectively). These data suggest that CSD modulation by daily estrogen injections in our study, where plasma levels peaked within an hour and declined to undetectable levels 12 h after the dose, is opposite to that of steady estrogen elevations for 2 weeks, highlighting the difficulty in pharmacologically recapitulating the ovarian cycle. Another study showed that topical application of β-estradiol in neocortical slices increased the repetition rate of CSDs induced by hypotonic medium [28]; however, obvious methodological differences preclude direct comparisons with our study. Importantly, we also studied castrated rats and did not find any effect of estrogen treatment or withdrawal, suggesting that genetic sex also plays a role in the CSD response to estrogens. It should be noted that all hormone treatment and withdrawal experiments were performed in gonadectomized animals in our study, precluding any interactions with other gonadal hormones.

Previous reports of enhanced CSD susceptibility upon prolonged high serum estradiol are also not congruent with the clinical risk of migraine attacks in relation to ovarian hormones [4, 5, 41]. For example, migraine attacks often improve during pregnancy when blood estradiol is persistently high. More importantly, menstrual migraine occurs just before and during menstruation, coincident with an abrupt drop in blood estradiol, implicating estrogen withdrawal rather than steady elevated levels as the culprit. Similarly, migraine frequency (with or without aura) increases between days 3–7 of the 7-day hormone-free interval in women treated with a combined hormonal contraception [42]. Indeed, the International Classification of Headache Disorders defines estrogen-withdrawal headache or migraine as a headache or a migraine developing within five days after the cessation of daily administration of exogenous estrogen for three weeks or longer and resolving within three days of its onset [43]. Importantly, this type of migraine occurs with daily oral contraceptives, which is associated with diurnal fluctuations in serum estrogen levels that we aimed to approximate [44, 45]. To date, there have been no clinical reports of estrogen-withdrawal migraine after estrogen patches or gels that are associated with steady estrogen elevations.

It is important to note that our estradiol replacement paradigm was not intended to recapitulate the hormonal changes during the normal menstrual cycle but rather approximated daily oral contraceptive use. As such, our estrogen withdrawal likely simulated the 7-day hormone-free interval in women treated with oral contraception rather than the premenstrual hormonal changes, and therefore, more relevant for estrogen-withdrawal migraines than menstrual migraines. Our daily estrogen replacement paradigm did create fluctuation of the plasma estradiol mimicking daily oral contraceptives, and CSD frequency increased seven days after the cessation of the 2-week treatment, congruent with estrogen-withdrawal migraine after oral contraceptive use. It is likely that the plasma half-life of estradiol does not reflect its biological half-life [33] since estradiol remains bound to estrogen receptors in the brain for at least 2 or 3 days [46, 47], and brain concentrations may remain high after treatment cessation, perhaps explaining the latency to an attack.

In contrast to estrogen, daily treatment with progesterone increased CSD frequency in this study. Others have reported a similar increase in CSD frequency after two week-implantation of progesterone capsules to Ovx rats or by single application to female rat neocortical slices [27, 28]. Progesterone and its metabolites have also been shown to enhance neuronal excitability [48, 49]. Therefore, experimental data suggest that progesterone may have an adverse effect on migraine pathophysiology, although clinical data on progesterone modulation of migraine are equivocal [41, 50].

To begin to address possible mechanisms contributing to reduced CSD susceptibility in ovariectomized rats and estrogen withdrawal-induced increased susceptibility, we used in vitro receptor autoradiography to examine the density of binding sites for sodium-dependent excitatory amino-acid transporters (the major transport mechanisms for extracellular glutamate removal in the central nervous system), for the 3 subtypes of ionotropic glutamate receptors and for GABA_A_ receptors. Although glutamatergic and GABAergic transmission are thought to underlie CSD mechanisms, migraine pathophysiology and mechanisms of action of prophylactic antimigraine agents [51], treatment-related changes in CSD susceptibility were not accompanied by changes in any of the binding sites examined. Several studies have used autoradiography to examine changes in receptor binding following ovariectomy and/or chronic estrogen treatment, including glutamate or GABA_A_. Such studies have reported increases in hippocampal NMDA receptor binding [52, 53], one study showing changes in female but not male rats [54], no effect on kainate or AMPA receptor density [53], and a decrease in GABA_A_ receptors in the frontal cortex [55]. The lack of a difference in NMDA or GABA_A_ binding among treatment groups in our study might be due to differences in treatment regimen or timing. Indeed, the effect of estradiol treatment on receptor densities can vary widely among studies. For example, both increased [56] and decreased [57, 58] [^3^H]muscimol binding following chronic administration have been reported. Nevertheless, these studies show that receptor autoradiography is sensitive enough to detect changes in receptor binding after interventions affecting female sex hormones. Of course, autoradiography only interrogates receptor binding rather than function. Possible changes at the level of receptor signal transduction and/or channel kinetics may have contributed to function. For example, chronic drug treatment may act downstream from receptors, leaving the density of binding sites unaffected [59]. Electrophysiological methods could more directly examine functional changes in glutamate or GABA receptor activity and could be undertaken in future studies.

As noted above, continuous topical KCl application to calculate the frequency of CSDs differs from CSD threshold determinations by stepwise escalating intensities of electrical stimulation or stepwise escalating concentrations of KCl as models for CSD susceptibility. While the former is indeed inversely, and presumably non-linearly, related to the CSD threshold, it is also influenced by changes in the absolute refractory period after a CSD. In theory, an intervention can influence CSD frequency by selectively altering the refractory period without actually changing the CSD threshold. Therefore, comparisons of our data to previous literature should consider this methodological difference. It should also be noted that other CSD attributes (speed, duration, amplitude) did not differ among any of the groups in this study. These are mainly used as quality control measures (i.e., experimental preparation, cortical integrity) and often lack the sensitivity to detect changes in CSD susceptibility.

## Conclusions

In summary, our data demonstrate the sexual dimorphism in CSD susceptibility. Higher CSD susceptibility in female rats did not differ among the estrous cycle stages, likely because of the short cycle duration compared with humans. More importantly, we show that estrogen withdrawal after a 2-week daily replacement paradigm in Ovx rats increased KCl-induced CSD frequency, mimicking estrogen-withdrawal migraines. More work is needed to elucidate the molecular and structural correlates of this enhanced CSD susceptibility. Of course, ovarian hormone modulation of migraine may also involve the nociceptive pathways [60], and mechanisms downstream to CSD should also be examined.

## Supplementary Information

**Additional file 1: Supplemental Table 1.** Specific parameters for the quantitative in vitro receptor labeling protocol for each radioligand.

**Additional file 2:** **Figure S1.** Effects of progesterone replacement and withdrawal in ovariectomized rats on CSD susceptibility. CSD frequency, speed, duration, and amplitude are shown for ovariectomized rats after 3 weeks of vehicle (Ovx+V, *n* = 12) or 5 mg/kg/day progesterone treatment (Ovx+P, *n* = 5) or progesterone treatment for 2 weeks followed by withdrawal (Ovx+P/W, *n* = 6).

## Data Availability

The database during and/or analyzed during the current study available from the corresponding author on reasonable request.

## Abbreviations

CSD: Cortical spreading depression
Cst: Castrated
DC: Direct current
KCl: Potassium chloride
NaCl: Sodium chloride
Ovx: Ovariectomized
rcf: Relative centrifugal force
SEM: Standard error of the mean

## References

[1] Jensen R, Stovner LJ (2008). Epidemiology and comorbidity of headache. Lancet Neurol.

[2] Sakai F, Igarashi H (1997). Prevalence of migraine in Japan: a nationwide survey. Cephalalgia.

[3] Gupta S, Mehrotra S, Villalon CM, Perusquia M, Saxena PR, MaassenVanDenBrink A (2007). Potential role of female sex hormones in the pathophysiology of migraine. Pharmacol Ther.

[4] Martin VT, Behbehani M (2006). Ovarian hormones and migraine headache: understanding mechanisms and pathogenesis–part 2. Headache.

[5] Martin VT, Behbehani M (2006). Ovarian hormones and migraine headache: understanding mechanisms and pathogenesis–part I. Headache.

[6] Martin VT, Lipton RB (2008). Epidemiology and biology of menstrual migraine. Headache.

[7] Ayata C, Lauritzen M (2015). Spreading depression, spreading depolarizations, and the cerebral vasculature. Physiol Rev.

[8] Kunkler PE, Kraig RP (2003). Hippocampal spreading depression bilaterally activates the caudal trigeminal nucleus in rodents. Hippocampus.

[9] Milner PM (1958). Note on a possible correspondence between the scotomas of migraine and spreading depression of Leao. Electroencephalogr Clin Neurophysiol.

[10] Lauritzen M, Jorgensen MB, Diemer NH, Gjedde A, Hansen AJ (1982). Persistent oligemia of rat cerebral cortex in the wake of spreading depression. Ann Neurol.

[11] Ayata C (2010). Cortical spreading depression triggers migraine attack: pro. Headache.

[12] Ayata C (2009). Spreading depression: from serendipity to targeted therapy in migraine prophylaxis. Cephalalgia.

[13] Hadjikhani N, Sanchez Del Rio M, Wu O, Schwartz D, Bakker D, Fischl B (2001). Mechanisms of migraine aura revealed by functional MRI in human visual cortex. Proc Natl Acad Sci U S A.

[14] Vincent MB, Hadjikhani N (2007). Migraine aura and related phenomena: beyond scotomata and scintillations. Cephalalgia.

[15] Zhang X, Levy D, Kainz V, Noseda R, Jakubowski M, Burstein R (2011). Activation of central trigeminovascular neurons by cortical spreading depression. Ann Neurol.

[16] Zhang X, Levy D, Noseda R, Kainz V, Jakubowski M, Burstein R (2010). Activation of meningeal nociceptors by cortical spreading depression: implications for migraine with aura. J Neurosci.

[17] Harriott AM, Chung DY, Uner A, Bozdayi RO, Morais A, Takizawa T (2021). Optogenetic spreading depression elicits trigeminal pain and anxiety behavior. Ann Neurol.

[18] Ayata C, Jin H, Kudo C, Dalkara T, Moskowitz MA (2006). Suppression of cortical spreading depression in migraine prophylaxis. Ann Neurol.

[19] Nozari A, Dilekoz E, Sukhotinsky I, Stein T, Eikermann-Haerter K, Liu C (2010). Microemboli may link spreading depression, migraine aura, and patent foramen ovale. Ann Neurol.

[20] Tamim I, Chung DY, de Morais AL, Loonen ICM, Qin T, Misra A (2021). Spreading depression as an innate antiseizure mechanism. Nat Commun.

[21] Eikermann-Haerter K, Dilekoz E, Kudo C, Savitz SI, Waeber C, Baum MJ (2009). Genetic and hormonal factors modulate spreading depression and transient hemiparesis in mouse models of familial hemiplegic migraine type 1. J Clin Invest.

[22] Balkaya M, Seidel JL, Sadeghian H, Qin T, Chung DY, Eikermann-Haerter K (2019). Relief following chronic stress augments spreading depolarization susceptibility in familial hemiplegic migraine mice. Neuroscience.

[23] Chauvel V, Multon S, Schoenen J (2018). Estrogen-dependent effects of 5-hydroxytryptophan on cortical spreading depression in rat: modelling the serotonin-ovarian hormone interaction in migraine aura. Cephalalgia.

[24] Brennan KC, Beltran-Parrazal L, Lopez-Valdes HE, Theriot J, Toga AW, Charles AC (2007). Distinct vascular conduction with cortical spreading depression. J Neurophysiol.

[25] Chauvel V, Vamos E, Pardutz A, Vecsei L, Schoenen J, Multon S (2012). Effect of systemic kynurenine on cortical spreading depression and its modulation by sex hormones in rat. Exp Neurol.

[26] Ebine T, Toriumi H, Shimizu T, Unekawa M, Takizawa T, Kayama Y (2016). Alterations in the threshold of the potassium concentration to evoke cortical spreading depression during the natural estrous cycle in mice. Neurosci Res.

[27] Chauvel V, Schoenen J, Multon S (2013) Influence of ovarian hormones on cortical spreading depression and its suppression by L-kynurenine in rat. PLoS One 8(12):e82279. 10.1371/journal.pone.0082279 10.1371/journal.pone.0082279 PMC3858280 24340013

[28] Sachs M, Pape HC, Speckmann EJ, Gorji A (2007). The effect of estrogen and progesterone on spreading depression in rat neocortical tissues. Neurobiol Dis.

[29] Adamek S, Vyskocil F (2011). Potassium-selective microelectrode revealed difference in threshold potassium concentration for cortical spreading depression in female and male rat brain. Brain Res.

[30] Negro A, Seidel JL, Houben T, Yu ES, Rosen I, Arreguin AJ (2020). Acute sleep deprivation enhances susceptibility to the migraine substrate cortical spreading depolarization. J Headache Pain.

[31] Kudo C, Nozari A, Moskowitz MA, Ayata C (2008). The impact of anesthetics and hyperoxia on cortical spreading depression. Exp Neurol.

[32] Skelton JL, Gardner-Medwin AR, George SA (1983). The effects of carbon dioxide, oxygen and pH on spreading depression in the isolated chick retina. Brain Res.

[33] Becker JB, Arnold AP, Berkley KJ, Blaustein JD, Eckel LA, Hampson E (2005). Strategies and methods for research on sex differences in brain and behavior. Endocrinology.

[34] Wong R, Ray D, Kendall DA (2012). Progesterone pharmacokinetics in the mouse: implications for potential stroke therapy. J Pharm Pharmacol.

[35] Ayata C (2013). Pearls and pitfalls in experimental models of spreading depression. Cephalalgia.

[36] Ajayi AF, Akhigbe RE (2020). Staging of the estrous cycle and induction of estrus in experimental rodents: an update. Fertil Res Pract.

[37] Brennan KC, Romero Reyes M, Lopez Valdes HE, Arnold AP, Charles AC (2007). Reduced threshold for cortical spreading depression in female mice. Ann Neurol.

[38] Charles A, Brennan K (2009). Cortical spreading depression-new insights and persistent questions. Cephalalgia.

[39] Westwood FR (2008). The female rat reproductive cycle: a practical histological guide to staging. Toxicol Pathol.

[40] Chai NC, Peterlin BL, Calhoun AH (2014). Migraine and estrogen. Curr Opin Neurol.

[41] MacGregor EA (2004). Oestrogen and attacks of migraine with and without aura. Lancet Neurol.

[42] Merki-Feld GS, Epple G, Caveng N, Imthurn B, Seifert B, Sandor P (2017). Temporal relations in hormone-withdrawal migraines and impact on prevention- a diary-based pilot study in combined hormonal contraceptive users. J Headache Pain.

[43] Headache Classification Committee of the International Headache Society (IHS) (2018). The International Classification of Headache Disorders, 3rd edition. Cephalalgia 38(1):1–211. 10.1177/0333102417738202. 10.1177/0333102417738202 29368949

[44] Todd C, Lagman-Bartolome AM, Lay C (2018). Women and migraine: the role of hormones. Curr Neurol Neurosci Rep.

[45] Edlow AG, Bartz D (2010). Hormonal contraceptive options for women with headache: a review of the evidence. Rev Obstet Gynecol.

[46] Schwartz SM, Blaustein JD, Wade GN (1979). Inhibition of estrous behavior by progesterone in rats: role of neural estrogen and progestin receptors. Endocrinology.

[47] Walker WA, Feder HH (1977). Anti-estrogen effects on estrogen accumulation in brain cell nuclei: neurochemical correlates of estrogen action on female sexual behavior in guinea pigs. Brain Res.

[48] Kapur J, Joshi S (2021) Progesterone modulates neuronal excitability bidirectionally. Neurosci Lett 744:135619. 10.1016/j.neulet.2020.135619 10.1016/j.neulet.2020.135619 PMC7821816 33421486

[49] Shiono S, Sun H, Batabyal T, Labuz A, Williamson J, Kapur J (2021). Limbic progesterone receptor activity enhances neuronal excitability and seizures. Epilepsia.

[50] Martin VT, Wernke S, Mandell K, Ramadan N, Kao L, Bean J (2005). Defining the relationship between ovarian hormones and migraine headache. Headache.

[51] Costa C, Tozzi A, Rainero I, Cupini LM, Calabresi P, Ayata C (2013). Cortical spreading depression as a target for anti-migraine agents. J Headache Pain.

[52] Woolley CS, Weiland NG, McEwen BS, Schwartzkroin PA (1997). Estradiol increases the sensitivity of hippocampal CA1 pyramidal cells to NMDA receptor-mediated synaptic input: correlation with dendritic spine density. J Neurosci.

[53] Weiland NG (1992). Estradiol selectively regulates agonist binding sites on the N-methyl-D-aspartate receptor complex in the CA1 region of the hippocampus. Endocrinology.

[54] Romeo RD, McCarthy JB, Wang A, Milner TA, McEwen BS (2005). Sex differences in hippocampal estradiol-induced N-methyl-D-aspartic acid binding and ultrastructural localization of estrogen receptor-alpha. Neuroendocrinology.

[55] Francois-Bellan AM, Segu L, Hery M (1989). Regulation by estradiol of GABAA and GABAB binding sites in the diencephalon of the rat: an autoradiographic study. Brain Res.

[56] Maggi A, Perez J (1984). Progesterone and estrogens in rat brain: modulation of GABA (gamma-aminobutyric acid) receptor activity. Eur J Pharmacol.

[57] Goetz C, Bourgoin S, Cesselin F, Brandi A, Bression D, Martinet M (1983). Alterations in central neurotransmitter receptor binding sites following estradiol implantation in female rats. Neurochem Int.

[58] Hamon M, Goetz C, Euvrard C, Pasqualini C, Le Dafniet M, Kerdelhue B (1983). Biochemical and functional alterations of central GABA receptors during chronic estradiol treatment. Brain Res.

[59] Pejchal T, Foley MA, Kosofsky BE, Waeber C (2002). Chronic fluoxetine treatment selectively uncouples raphe 5-HT(1A) receptors as measured by [(35)S]-GTP gamma S autoradiography. Br J Pharmacol.

[60] Martin VT, Lee J, Behbehani MM (2007). Sensitization of the trigeminal sensory system during different stages of the rat estrous cycle: implications for menstrual migraine. Headache.

