# Supplementary material for: Estrogen modulation of cortical spreading depression
Source: J Headache Pain. 2023 May 26;24(1):62. doi: 10.1186/s10194-023-01598-x (PMC10214707; doi:10.1186/s10194-023-01598-x)
Supplement: Supplementary file 1 — Additional file 1: Supplemental Table 1. Specific parameters for the quantitative in vitro receptor labeling protocol for each radioligand. [file 10194_2023_1598_MOESM1_ESM.docx]

**Supplemental Table 1: Specific parameters for the quantitative in vitro receptor labeling protocol for each radioligand.**

| **Radioligand** | **Nonspecific binding** | **Preincubation** | **Incubation** | **Wash** | **Exposure time** |
| --- | --- | --- | --- | --- | --- |
| D-[^3^H]aspartate (88 nM) | 250 µM D-Aspartic acid | 60 min at 4°C in 50 mM Tris·HCl pH 7.4 | 30 min at 4°C in 50 mM Tris·HCl pH 7.4  300 mM NaCl | - 4x5sec in cold buffer - Dip in cold water - 5 seconds in acetone with 2.5% glutaraldehyde | 2 weeks |
| [^3^H]kainate  (12 nM) | 30 µM kainic acid | 45 min at 4°C in 50 mM Tris·Acetate pH 7.2 | 45 min at 4°C in 50 mM Tris·Acetate pH 7.2 | - 3x10 sec in cold buffer - 5 seconds in acetone with 2.5% glutaraldehyde | 2 weeks |
| [^3^H]AMPA  (20 nM) | 30 µM CNQX (6-cyano-7-nitroquinoxaline-2,3-dione) | 45 min at 4°C in 50 mM Tris·Acetate pH 7.2 | 45 min at 4°C in 50 mM Tris·Acetate pH 7.2, 100 mM KSCN | - 3x3 sec in cold buffer - 5 seconds in acetone with 2.5% glutaraldehyde | 1 week |
| [^3^H]MK-801  (5 nM) | 100 µM MK-801 | 30 min at 4°C in 5 mM Tris·HCl pH 7.4 | 60 min at room temperature in 5 mM Tris·HCl pH 7.4, 50 µM L-glutamate, 50 µM spermidine, 50 µM glycine | - 30 sec and 2x5 min in cold 5 mM Tris·HCl pH 7.4 - Dip in cold water | 1 week |
| [^3^H]muscimol  (8 nM) | 300 µM GABA | 2x10 min at 4°C in 50 mM Tris·Citrate pH 7.1 | 40 min at 4°C in 50 mM Tris·Citrate pH 7.1 | - 3x15 sec and 2x5 min in cold buffer - Dip in cold water | 2 weeks |
