# Supplementary figures and images for: Estrogen modulation of cortical spreading depression
Source: J Headache Pain. 2023 May 26;24(1):62. doi: 10.1186/s10194-023-01598-x (PMC10214707; doi:10.1186/s10194-023-01598-x)

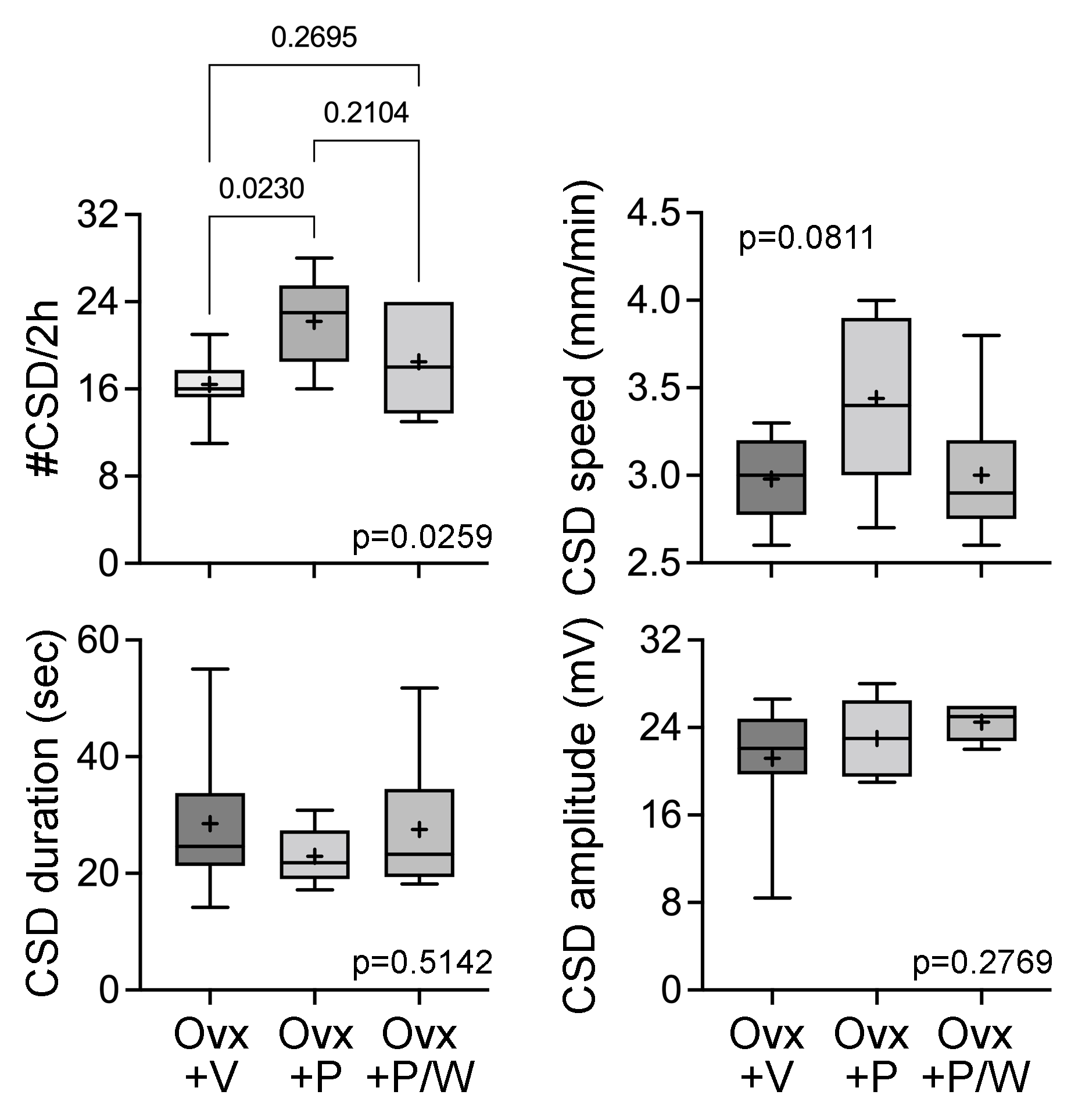

Supplement: Supplementary file 2 — Additional file 2: Figure S1. Effects of progesterone replacement and withdrawal in ovariectomized rats on CSD susceptibility. CSD frequency, speed, duration, and amplitude are shown for ovariectomized rats after 3 weeks of vehicle (Ovx+V, n = 12) or 5 mg/kg/day progesterone treatment (Ovx+P, n = 5) or progesterone treatment for 2 weeks followed by withdrawal (Ovx+P/W, n = 6). [file 10194_2023_1598_MOESM2_ESM.tif]
